# Artificial intelligence-assisted colonoscopy for colorectal lesion detection: a case-control study on diagnostic accuracy and histopathological agreement

**DOI:** 10.1590/0102-67202025000029e1898

**Published:** 2025-09-08

**Authors:** Marcio Roberto FACANALI, Afonso Henrique da Silva SOUSA, Carlos Frederico Sparapan MARQUES, Adriana Vaz SAFATLE-RIBEIRO

**Affiliations:** 1 Universidade de São Paulo, Faculty of Medicine, Department of Gastroenterology, Colonoscopy Division – São Paulo (SP), Brazil.

**Keywords:** Artificial intelligence, Colonoscopy, Adenoma, Colorectal neoplasms, Early detection of cancer, Inteligência artificial, Colonoscopia, Adenoma, Neoplasias colorretais, Detecção precoce de cancer

## Abstract

**Background:**

Artificial intelligence (AI)-assisted colonoscopy has emerged as a tool to enhance adenoma detection rates (ADRs) and improve lesion characterization. However, its performance in real-world settings, especially in developing countries, remains uncertain.

**Aims:**

The aim of this study was to evaluate the impact of AI on ADRs and its concordance with histopathological diagnosis.

**Methods:**

A matched case–control study was conducted at a colorectal cancer (CRC) referral center, including 146 patients aged 45–75 years who underwent colonoscopy for CRC screening or surveillance. Patients were allocated into two groups: AI-assisted colonoscopy (n=74) and high-definition conventional colonoscopy (n=72). The primary outcome was ADR, and the secondary outcome was the agreement between AI-based lesion characterization and histopathology. Statistical analysis was performed with a significance level of p<0.05.

**Results:**

ADR was higher in the AI group (60%) than in the control group (50%), but this difference was not statistically significant (p>0.05). AI-assisted lesion characterization showed substantial agreement with histopathology (kappa=0.692). No significant difference was found in withdrawal time (29 min vs. 27 min; p>0.05), indicating that AI did not delay the procedure

**Conclusions:**

Although AI did not significantly increase ADR compared to conventional colonoscopy, it demonstrated strong histopathological concordance, supporting its reliability in lesion characterization. AI may reduce interobserver variability and optimize real-time decision-making, reinforcing its clinical utility in CRC screening.

## ARTICLE HIGHLIGHTS

- Lesion characterization by artificial intelligence (AI) demonstrated substantial agreement with histopathological findings, supporting its diagnostic reliability.
- Despite no significant increase in adenoma detection rates (ADRs), AI proved valuable in reducing interobserver variability and enhancing real-time clinical decision-making during colorectal cancer screening.
- AI integration did not significantly prolong withdrawal time (29 vs. 27 min; p>0.05), indicating its seamless incorporation into the clinical workflow.
- AI-assisted colonoscopy showed a higher ADR (60%) compared to conventional colonoscopy (50%), though the difference was not statistically significant.

## CENTRAL MESSAGE

Screening strategies, particularly colonoscopy, play a vital role in identifying and removing precancerous lesions before they develop into invasive cancer, significantly improving patient outcomes. Recent technological advancements have introduced artificial intelligence (AI)-assisted colonoscopy as a promising tool to enhance detection accuracy. AI-based computer-aided detection (CADe) systems have demonstrated the potential to improve adenoma detection rates (ADRs) by identifying subtle polyps that might otherwise be overlooked by endoscopists.

## PERSPECTIVES

Artificial intelligence (AI)-assisted colonoscopy has demonstrated strong concordance with histopathological findings, reinforcing its reliability in lesion characterization. Additionally, AI has shown the potential for enhancing diagnostic consistency, reducing interobserver variability, and optimizing real-time decision-making in colorectal cancer (CRC) screening. Its minimal impact on withdrawal time further supports its feasibility and seamless integration into routine clinical practice.

## VISUAL ABSTRACT

**Figure:**
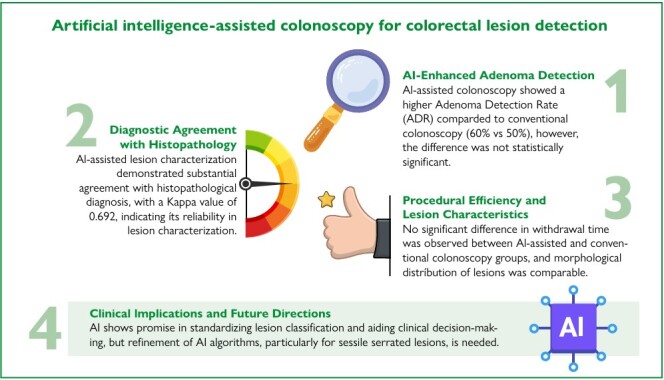

## RESUMO

**Racional:**

A colonoscopia assistida por inteligência artificial (IA) tem sido proposta como uma ferramenta para aumentar a taxa de detecção de adenomas (TDA) e melhorar a caracterização de lesões. No entanto, sua eficácia em ambientes clínicos reais, especialmente em países em desenvolvimento, ainda é incerta.

**Objetivos:**

Avaliar o impacto da IA sobre a TDA e sua concordância com o diagnóstico histopatológico.

**Métodos:**

Estudo caso-controle pareado realizado em um centro de referência para rastreamento de câncer colorretal (CCR), incluindo 146 pacientes entre 45 e 75 anos submetidos à colonoscopia para rastreamento ou vigilância de CCR. Os pacientes foram alocados em dois grupos: colonoscopia com IA (n=74) e colonoscopia convencional de alta definição (n=72). O desfecho primário foi a TDA, e o secundário, a concordância entre a caracterização da lesão por IA e o resultado histopatológico. O nível de significância foi p<0,05.

**Resultados:**

A TDA foi maior no grupo com IA (60%) do que no controle (50%), sem significância estatística (p>0,05). A caracterização das lesões pela IA mostrou concordância substancial com a histopatologia (Kappa=0,692). O tempo de retirada do colonoscópio não diferiu significativamente entre os grupos (29 min vs. 27 min; p>0,05), indicando que a IA não interferiu na eficiência do procedimento.

**Conclusões:**

Embora não tenha aumentado significativamente a TDA, a IA demonstrou alta concordância histológica, reforçando sua utilidade na caracterização de lesões e na tomada de decisão clínica em tempo real.

## INTRODUCTION

 Colorectal cancer (CRC) remains a major global health challenge, accounting for a substantial number of cancerrelated deaths annuallysup^23^. The progression of CRC typically follows a multistep process, evolving from benign adenomatous polyps to malignant lesions, emphasizing the critical need for early detection and interventionsup^4,13^. 

 Screening strategies, particularly colonoscopy, play a vital role in identifying and removing precancerous lesions before they develop into invasive cancer, significantly improving patient outcomes^15^. Despite its efficacy, traditional colonoscopy remains operator-dependent, with lesion detection rates varying among endoscopists, leading to the potential for missed adenomas, especially flat or subtle neoplastic lesions^8 ,12,18^ . 

 Recent technological advancements have introduced artificial intelligence (AI)-assisted colonoscopy as a promising tool to enhance detection accuracy. AI-based computer-aided detection (CADe) systems have demonstrated the potential to improve adenoma detection rates (ADRs) by identifying subtle polyps that might be overlooked by endoscopists^27^. Studies indicate that AI algorithms excel at detecting non-polypoid lesions and small adenomas, which are often challenging to identify during standard colonoscopy^1^. Furthermore, AI integration into real-time colonoscopy has been associated with increased sensitivity in detecting high-risk neoplasms, bridging the gap in conventional diagnostic approaches^21^. 

 Despite these promising findings, the impact of AI-assisted colonoscopy varies across different populations and healthcare systems. While some studies report significant improvements in ADRs with AI, others suggest that its greatest benefit lies in reducing interobserver variability and enhancing the detection of diminutive polyps rather than increasing the identification of advanced colorectal neoplasia^5^. Moreover, most of the existing research has been conducted in high-income countries, leaving a gap in the literature regarding the effectiveness of AIassisted colonoscopy in developing regions, where CRC screening programs may differ in accessibility and implementation^22^. 

 As AI-driven endoscopic technologies are predominantly studied in high-income settings and their applicability and effectiveness in resource-limited healthcare settings remain unclear, this study aims to evaluate the ADR of AI-assisted colonoscopy in such settings. Furthermore, it seeks to assess the accuracy of AI in characterizing lesions compared to histopathological findings. By addressing this gap, this research aims to provide valuable insights into the potential benefits and limitations of integrating AI into CRC screening and diagnosis in developing countries. 

## METHODS

### Study design

 A single-center, retrospective, matched case–control observational study was proposed, conducted at a reference center for CRC screening and surveillance in São Paulo, Brazil. This study was submitted to the Brazilian Research Ethics Platform (Plataforma Brazil) and approved by the Ethical Committee of the institution (CAAE nº 87207125.0.0000.0068). 

### Population

 Patients aged 45–75 years who underwent colonoscopy for CRC screening or surveillance from June 2021 to June 2023 were selected. 

#### Inclusion criteria

 Patients were eligible for inclusion in the study if they met the following criteria:

- Age between 45 and 75 years.
- Having undergone a colonoscopy for CRC screening or surveillance during the study period.
- Examinations that were performed by experienced colonoscopists (each with experience of more than 3000 colonoscopies).

#### Exclusion criteria

The following were excluded from the study:

- Patients with incomplete colonoscopies, i.e., those that did not reach the cecum, for any reason.
- Patients with a history of any partial or total colectomy.
- Patients diagnosed with inflammatory bowel disease.
- Cases involving lesions that could not be adequately characterized by AI, preventing cross-referencing with histopathological analysis.

#### Procedures

Patients were divided into two study groups:

- Control group: Colonoscopies performed without AI assistance, with Fujifilm 6000 and 7000 series colonoscopes and the Eluxeo 7000 processor (Fujifilm, Tokyo, Japan), where lesion identification was conducted exclusively by the colonoscopist.
- Case group: Colonoscopies performed with the Fujifilm 7000 series colonoscope, the Eluxeo 7000 processor, and the CAD EYE AI system (Fujifilm, Tokyo, Japan), which aid in the detection and characterization of colorectal lesions. All lesions characterized by AI were resected in separate vials, and the findings detected by AI were then compared with the results of histopathology, which served as the gold standard.

### Study objectives

#### Primary outcome

To compare the colorectal ADR between the two groups, with a particular focus on adenomatous lesions.

#### Secondary outcomes

To assess the diagnostic agreement between lesions detected by AI and histopathology.

To calculate the sensitivity, specificity, positive predictive value (PPV), and negative predictive value (NPV) of AI in the detection of colorectal lesions.

To determine the kappa coefficient to assess the level of agreement between the findings of AI and histopathology.

### Statistical analysis

 The quantitative characteristics of the patients were described using mean, standard deviation (SD), median, and quartiles, and were compared between groups using the following statistical tests: unpaired Student’s t-test for normally distributed variables and Mann-Whitney U test for non-normally distributed variables. Qualitative variables were described using absolute and relative frequencies, and associations between groups were analyzed using the chi-square test and likelihood ratio test. Diagnostic agreement between AI and histopathology was assessed using the kappa coefficient, and diagnostic measures (sensitivity, specificity, PPV, and NPV) were calculated with 95% confidence intervals (CIs), considering histopathology as the gold standard. 

 All statistical analyses were performed using IBM SPSS for Windows, version 22.0, and data were tabulated using Microsoft Excel 2013. The significance level was set at 5% (p<0.05). 

## RESULTS

 A total of 146 patients were included in the study, of whom 74 underwent AI-assisted colonoscopy (case group) and 72 underwent high-definition colonoscopy without AI assistance (control group). Both groups exhibited a predominance of female patients (68.9% in the case group vs. 68.1% in the control group). 

 As shown in Table 1, the mean age was significantly higher in the control group compared to the AI-assisted group (p=0.024 vs. p>0.05). Additionally, the frequency of a family history of CRC was significantly greater in the control group compared to the AI-assisted group (p=0.026 vs. p>0.5). However, there were no significant differences (p>0.05) between the groups with regard to other baseline characteristics, particularly regarding the number and type of identified lesions. 

**Table 1:** Patient characteristics by group and statistical test results

Variables		Control group	Case group	Total	p-value
Age (years)					
	Mean±SD	62.1±8	59.1±7.8	60.6±8	0.024*
	Median (p25; p75)	62 (56; 68)	60 (52.5; 67)	61 (54; 67)	
Sex (%)					
	Female	49 (68.1)	52 (69.3)	101 (68.7)	0.867
	Male	23 (31.9)	23 (30.7)	46 (31.3)	
Smoker (%)					
	No	57 (80.3)	57 (80.3)	114 (80.3)	0.319
	Yes	9 (12.7)	5 (7)	14 (9.9)	
	Former smoker	5 (7)	9 (12.7)	14 (9.9)	
Family history (%)					
	No	46 (63.9)	58 (80.6)	104 (72.2)	**0.026†**
	Yes	26 (36.1)	14 (19.4)	40 (27.8)	
Withdrawal time (minutes)					
	Mean±SD	27±9.8	29.1±10	28±9.9	0.137‡
	Median (p25; p75)	25 (20; 33)	28 (22; 35)	28 (22; 33)	
Total lesions detected					
	Mean±SD	2.33±1.23	2.23±1.48	2.27±1.37	0.408‡
	Median (p25; p75)	2 (1; 3)	2 (1; 3)	2 (1; 3)	
Adenomas detected					
	Mean±SD	1.89±1.04	1.73±0.95	1.8±0.99	0.481‡
	Median (p25; p75)	2 (1; 2.8)	1 (1; 2)	1 (1; 2)	
Serrated lesions detected					
	Mean±SD	2±1	1.5±1	1.6±0.89	0.429‡
	Median (p25; p75)	2 (2; 2)	1 (1; 2.5)	1 (1; 2.5)	

χ^2^ test. Statistically significant values are denoted in bold. SD: standard deviation.

* Unpaired Student’s t-test

† Likelihood ratio test

‡ Mann-Whitney test; and some cases lack information

### Adenoma detection rate and procedural aspects

 The ADR was higher in the AI-assisted group (60%) compared to the control group (50%) in absolute numbers; however, this difference was not statistically significant (p>0.05), as shown in Table 2. 

**Table 2:** Description of the characteristics of the lesions found according to groups and results of statistical tests.

Variables		Control group (%)	Case group (%)	Total (%)	p-value
Lesion location					
	Cecum	14 (13.1)	6 (4.8)	20 (8.6)	0.079*
	Ascending colon	28 (26.2)	25 (20)	53 (22.8)	
	Hepatic flexure	5 (4.7)	12 (9.6)	17 (7.3)	
	Transverse colon	17 (15.9)	19 (15.2)	36 (15.5)	
	Splenic flexure	2 (1.9)	0 (0)	2 (0.9)	
	Descending colon	8 (7.5)	13 (10.4)	21 (9.1)	
	Sigmoid colon	17 (15.9)	24 (19.2)	41 (17.7)	
Rectum		16 (15)	26 (20.8)	42 (18.1)	
Histopathology					
	Adenoma with low-grade dysplasia	65 (60.7)	75 (60)	140 (60.3)	0.428*
	Adenoma with high-grade dysplasia	2 (1.9)	1 (0.8)	3 (1.3)	
	Serrated	1 (0.9)	5 (4)	6 (2.6)	
	Serrated with low-grade dysplasia	1 (0.9)	1 (0.8)	2 (0.9)	
	Inflammatory	3 (0.9)	0 (0)	3 (0.9)	
	Hyperplastic	36 (33.6)	43 (34.4)	79 (34.1)	
	Well-differentiated adenocarcinoma	1 (0.9)	0 (0)	1 (0.4)	
Lesion size (mm)					
	Mean±SD	4.36±3.81	4.69±4.19	4.55±4.02	**0.537†**
	Median (p25; p75)	3 (2; 4)	3 (2; 5)	3 (2; 4.8)	

Statistically significant values are denoted in bold. SD: standard deviation.

* Likelihood ratio test

† Mann–Whitney test

 Regarding procedural efficiency, the mean time to withdraw the colonoscope was 29 min (SD: 9.97) in the case group compared to 27 min (SD: 9.8) in the control group; however, this difference was not statistically significant (p=0.137, p>0.05). 

### Lesion characteristics and morphological distribution

 The morphological distribution of the identified lesions was comparable between the groups (p>0.05). Both groups presented similar rates of serrated lesions, adenomas, and hyperplastic polyps. 

 Lesion characterization provided by AI showed substantial concordance with histopathological diagnosis (kappa=0.692), as shown in Table 3. AI exhibited strong sensitivity and specificity, with a high PPV. However, the NPV was slightly lower, indicating a moderate rate of false-negative findings. 

**Table 3:** Cross-referencing of artificial intelligence and histopathological diagnostic results and results of the agreement coefficient and diagnostic measures for identifying neoplastic lesions.

AI diagnosis	Histopathological diagnosis		Total (95%CI)	Kappa (95%CI)	Sensitivity (%) (95%CI)	Specificity (%) (95%CI)	PPV (%) (95%CI)	NPV (%) (95%CI)
	Neoplastic (%)	Hyperplastic (%)						
Neoplastic	67 (54.5)	4 (3.3)	71 (57.7)	0.692 (0.563–0.821)	82.7 (72.7–90.2)	90.5 (77.4–97.23)	94.4 (86.2–98.4)	73.1 (59–84.4)
Hyperplastic	14 (11.4)	38 (30.9)	52 (42.3)					
Total	81 (65.9)	42 (34.1)	123 (100)					

AI: artificial intelligence; Cl: confidence interval; PPV: positive predictive value; NPV: negative predictive value.

## DISCUSSION

 In this study, a total of 146 patients were evaluated, of whom 74 underwent AI-assisted colonoscopy (case group) and 72 underwent high-definition colonoscopy without AI assistance (control group). The two groups were demographically comparable except for age and family history of CRC, which were significantly higher in the control group (p=0.024 and p=0.026, p>0.05, respectively). 

 In this study, the mean colonoscope withdrawal time was 29 min in the AI-assisted group and 27 min in the control group, with no statistically significant difference (p>0.05), supporting the idea that AI can improve lesion detection without introducing delays to the procedure. Studies suggest that the learning curve for AI-assisted colonoscopy is relatively short, meaning that experienced endoscopists can quickly integrate AI prompts into their workflow^3^. 

 AI-assisted lesion characterization showed substantial agreement with histopathological diagnoses (kappa=0.692), reinforcing its potential as a reliable diagnostic tool, suggesting that it effectively distinguishes neoplastic from non-neoplastic lesions. These results are consistent with previous studies reporting high concordance rates between AI-based optical diagnosis and histopathology^24^. 

 Similar findings have been reported in systematic reviews, where AI-assisted colonoscopy showed more than 90% concordance with histopathological results^25^. This high level of concordance supports the usefulness of AI in real-time lesion characterization, reducing the need for unnecessary resections and optimizing clinical decision-making. 

 Real-time lesion diagnosis can reduce interobserver variability, especially in centers with different levels of expertise, optimizing decision-making at the time of examination and helping endoscopists in deciding whether a lesion requires resection and which technique would be most appropriate for that lesion. 

 In addition, AI systems could improve the cost-effectiveness of CRC screening by reducing unnecessary biopsies and resections, especially in developing countries where financial resources are limited. A study by Young et al.^28^, published in 2023, showed that AI-guided optical diagnosis can support "resection and discard" strategies, reducing histopathology costs without compromising accuracy. 

 On the other hand, the ADR was 60% in the AI-assisted group compared to 50% in the control group. Although the absolute ADR was higher in the AI group, this difference was not statistically significant (p>0.05). 

 In this study, all procedures were performed by experienced endoscopists, each with experience in performing more than 3,000 colonoscopies. Repici et al.^20^, demonstrated in a randomized controlled clinical trial that AI significantly improved lesion detection rates among non-expert endoscopists (less than 2,000 colonoscopies) when compared to previously published data from experienced endoscopists. However, the impact of AI was less pronounced when used by highly experienced operators. 

 In contrast to some systematic reviews, such as that of Hassan et al.^11^, which analyzed five clinical randomized trials and demonstrated a significantly higher ADR in the AI-assisted group compared to the control group (36.6% vs. 25.2%; relative risk [RR]: 1.44; 95%CI 1.27–1.62), a more recent systematic review by Wei et al.^26^, published in 2024, evaluating non-randomized studies assessing AI-assisted colonoscopy in real-world settings, demonstrated higher ADR only in prospective studies. Their findings indicated that AI-assisted colonoscopy achieved a statistically higher ADR (36.3 vs. 35.8%; RR 1.13; 95%CI 1.01–1.28), however, with no statistically significant difference in retrospective studies, which may help explain the results of the present study, as it was retrospective in nature. 

 To further investigate the discrepancies in findings across systematic reviews, Pan et al. analyzed seven discordant meta-analyses on AI-assisted colonoscopy and concluded that this technology has the potential to improve the detection of polyps and adenomas^17^. Similarly, Makar et al. also demonstrated that AI-assisted colonoscopy significantly enhances adenoma detection^16^. 

 We believe that another factor contributing to the lack of superiority of AI over standard high-definition colonoscopy in this study was the difference in age (p=0.024 vs. p< 0.05) and a family history of CRC (p=0.026 vs. p< 0.05), both of which were statistically higher in the standard colonoscopy group (control group) than in the AI group. Although these differences may not completely invalidate the comparisons, they warrant careful interpretation of the findings, as they introduce potential confounding factors or bias. It is well established that older patients may have undergone more frequent CRC screening and surveillance, potentially influencing lesion detection rates. Studies suggest that adenoma prevalence increases with age, and older patients are more likely to have undergone prior polypectomies or colonoscopic evaluations^2^. In contrast, younger patients in the AI-assisted group may have a lower adenoma burden, potentially underestimating the true efficacy of AI in high-risk populations. 

 Furthermore, patients with a family history of CRC may have a higher baseline risk of developing precancerous or malignant lesions, leading to increased lesion detection rates regardless of AI use. Prior research has demonstrated that individuals with a family history of CRC have a higher ADR regardless of the screening method employed^10^. The imbalance in both age and family history between the study groups may have artificially diminished the apparent impact of AI, necessitating cautious interpretation of these results. 

 Fujifilm’s CAD EYE system, the AI technology used in this study, demonstrated high accuracy in differentiating neoplastic from hyperplastic lesions, but one of its main limitations was the misclassification of serrated lesions as hyperplastic. Because CAD EYE primarily classifies lesions into neoplastic and hyperplastic categories, serrated lesions without dysplasia are often categorized as hyperplastic polyps, as some studies have shown^6,7^. This misclassification is particularly problematic because sessile serrated lesions (SSLs) are precancerous and require different surveillance strategies compared to truly hyperplastic lesions^14^. 

 De Lange et al.^6^, in a prospective multicenter study, demonstrated that AI-assisted colonoscopy systems, including CAD EYE, showed improved accuracy in conventional adenoma detection, but their performance in differentiating SSLs from hyperplastic polyps remained inconsistent. Given that SSLs contribute significantly to interval CRC, improving the ability of AI to correctly classify these lesions is crucial for effective CRC prevention. 

 Incorporating advanced deep learning models trained on a larger dataset of serrated lesions may ensure better differentiation of hyperplastic lesions in the future, just as the integration of multimodal imaging techniques, such as narrow-band imaging or texture-based analysis (chromoendoscopy), may refine lesion classification and reduce misclassification rates^9,19^. 

 As strengths, we highlight the real-world applicability of AI, as this study was conducted in a high-volume referral center rather than a strictly controlled experimental setting, thereby reflecting real-world clinical conditions. 

 The case-control design allows for a direct comparison between AI-assisted colonoscopy (CAD EYE) and conventional high-definition colonoscopy, providing significant insights into the added value of AI. AI-assisted diagnoses were systematically cross-referenced with histopathology results, ensuring that lesion classification was confirmed by the gold standard. 

 While this study provides valuable insights into the effectiveness of AI-assisted colonoscopy, limitations should be considered, such as the study being conducted in a single referral center, which may limit generalizability to other healthcare settings. Also, the non-randomized nature of case-control studies introduces the possibility of selection bias. Additionally, in this study, the control group had a higher mean age and a higher frequency of family history of CRC, which could influence ADR, potentially masking the true impact of AI. Another point is that this study evaluated immediate lesion detection and concordance with histopathology but did not evaluate long-term clinical outcomes, such as the incidence of interval CRC or recurrence rates after polypectomy. Finally, although AI-assisted colonoscopy demonstrated high concordance with histopathology, the study design and methodological limitations may have influenced the findings. 

 Although AI did not significantly improve ADR compared with conventional high-definition colonoscopy, its role in standardizing lesion classification and assisting clinical decision-making remains promising. Future research should focus on refining AI algorithms, particularly in the classification of SSLs, and assessing their benefits across different levels of endoscopist experience. As AI technology continues to evolve, its potential to enhance procedural efficiency and diagnostic accuracy warrants further investigation. 

 Addressing these limitations through multicenter validation, randomized trials, and long-term follow-up will be essential to fully establish the role of AI in CRC screening and surveillance. 

## CONCLUSIONS

 AI-assisted colonoscopy has demonstrated strong concordance with histopathological findings, reinforcing its reliability in lesion characterization. Additionally, AI has shown the potential for enhancing diagnostic consistency, reducing interobserver variability, and optimizing real-time decision making in CRC screening. Its minimal impact on withdrawal time further supports its feasibility and seamless integration into routine clinical practice. 

## Data Availability

The information regarding the investigation, methodology, and data analysis of the article is archived under the responsibility of the authors.
